# Alternative treatment for patent ductus arteriosus: a therapeutic challenge

**DOI:** 10.1590/1677-5449.210085

**Published:** 2021-09-03

**Authors:** Lauro Henrique Heinsch Domenighi, Guilherme Pinto Quoos, Stela Karine Braun, Alcides André Dezordi Vogel, Romualdo Bolzani dos Santos, Vinícius Matos Menegola

**Affiliations:** 1 Universidade Franciscana – UFN, Santa Maria, RS, Brasil.; 2 Hospital Universitário de Santa Maria – HUSM, Santa Maria, RS, Brasil.; 3 Universidade Federal de Santa Maria – UFSM, Santa Maria, RS, Brasil.

**Keywords:** ductus arteriosus patent, endovascular procedures, vascular surgical procedures, canal arterial persistente, procedimentos endovasculares, procedimentos cirúrgicos vasculares

## Abstract

The ductus arteriosus is a fetal structure that spontaneously closes in 90% of newborns. Patency 3 months after birth is considered a congenital heart disease that, if untreated, can progress to serious cardiovascular complications. This report aims to review an alternative treatment with an aortic endoprosthesis in a 49-year-old man who presented with dyspnea on moderate exertion associated with a heart murmur. He was diagnosed with persistent ductus arteriosus (PDA) with cardiac complications. Clinical management was unsuccessful and surgical treatment was indicated. Endovascular treatment with a thoracic endoprosthesis was indicated and performed successfully. Percutaneous closure is the preferred method in adult patients. Endovascular intervention using an endoprosthesis is a safe and effective option, in addition to being applicable regardless of the anatomy of the PDA. This case demonstrates the natural history of the pathology and presents a safe and effective alternative for its management.

## Resumo

O canal arterial é uma estrutura fetal que se fecha espontaneamente em 90% dos recém-nascidos. A permeabilidade por mais de 3 meses é considerada uma cardiopatia congênita que, se não tratada, pode evoluir para complicações cardíacas graves. Este trabalho relata um tratamento alternativo com endoprótese aórtica. Trata-se de um homem, 49 anos, que apresentou dispneia aos moderados esforços, associada com um sopro cardíaco. Foi diagnosticado com canal arterial persistente (PCA) já com complicações cardíacas. Tentou-se manejo clínico sem sucesso, sendo indicado tratamento cirúrgico. Optou-se pelo tratamento endovascular com uma endoprótese torácica, o qual foi realizado com sucesso. O fechamento percutâneo é o método preferido em pacientes adultos. A intervenção endovascular com o uso da endoprótese é uma possibilidade segura e eficaz, além de ser aplicável independentemente da anatomia do PCA. Este caso, além de demonstrar a história natural desta patologia, apresenta uma alternativa segura e eficaz empregada no seu manejo.

## INTRODUCTION

Ductus arteriosus is a fetal vascular structure that connects the left pulmonary artery with the descending aorta.^1^^-^5 It closes spontaneously in up to 90% of full-term newborns during their first 48 hours of life. Patency lasting more than 3 months is considered a congenital heart disease. The incidence in full-term children is 1 case in 2000 live births. However, it is present in 65% of those born weighing 1000g or less and, in this situation, it is associated with several morbidities. The ratio between the sexes is 2 female cases to 1 male case.^1^

An untreated persistent ductus arteriosus (PDA) can progress to several clinical complications such as Atrial Fibrillation (AF); ventricular hypertrophy with congestive heart failure; pulmonary vascular disease; inadequate physical development; infective endocarditis; and aneurysmal dilatation of the duct and calcification.^2^^,^^6^^,^^7^

In the case described, the possibility of endovascular treatment using an aortic endoprosthesis was demonstrated. This approach can be employed regardless the size of the PDA and is safe and effective.^8^^-^11 This study was approved by the Research Ethics Committee at the Federal University of Santa Maria (CEP-UFSM). Ruling number 3.326.266. CAE:2989119.0.0000.5346.

## Part I – CLINICAL SITUATION

A 49-year-old male patient with a history of hypertension sought medical services for dyspnea on moderate exertion with relief at rest, associated with a heart murmur. Previous transthoracic echocardiographs (TTE) showed persistence of the ductus arteriosus (PDA), with significant volumetric overload of left atrium and ventricle, moderate aortic ectasia, minimal aortic valve insufficiency, and mild eccentric hypertrophy of the left ventricle.

**Figure 1:**
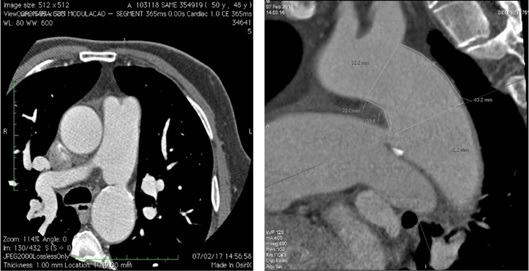
Angiotomography sections showing Krichenko B-type PDA.

A new TTE revealed moderate left ventricular hypertrophy, AF, pulmonary arterial hypertension with a pressure of 44 mmHg (not an exact measurement because of the PDA), and an ejection fraction of 50%. Cardiac catheterization revealed mild pulmonary hypertension, average 25 mmHg, and presence of significant shunt at the level of the pulmonary artery, estimating a Qs / Qp > 2.5l / min, without obstructive coronary lesions. Aortic angiotomography classified the PDA as Krichenko type B, associated with ectasia of the pulmonary artery trunk and its central branches, and enlargement of the cardiac chambers, which can be seen in Figure 1. After 5 months of follow-up, his symptoms had worsened.

## Part II – WHAT WAS DONE

After evaluation by cardiac and vascular surgery teams, endovascular treatment was indicated due to structural changes that represented increased risk from cardiopulmonary bypass. Also, the University Hospital did not have the necessary plugs for PDA embolization and the patient’s anatomy was unfavorable for this technique. Endovascular treatment was performed.

**Figure 2:**
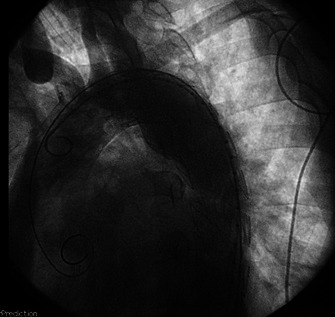
Control angiography that demonstrated good stent positioning, absence of leaks, and patency of the left common carotid artery, left subclavian artery, and brachiocephalic trunk.

The procedure started with retrograde puncture and insertion of a 5Fr introducer into the left common femoral artery. The right common femoral artery was dissected and a 5Fr introducer placed. This was followed by passage of a 0.035” hydrophilic guide and pigtail catheter (PIG) centered on the left common femoral artery and then an aortography was performed, which showed the PDA. The Lunderquist guide was continued through the right common femoral artery to the ascending aorta, followed by implantation of a Zenith Alpha endoprosthesis (44x179mm) in zone 3 of the aortic arch and descending aorta. Control angiography showed absence of leaks and patency of the left common carotid artery, left subclavian artery, and brachiocephalic trunk (Figure 2). The patient had a good postoperative recovery without complications in the Cardiac Intensive Care Unit, where he remained for 3 days. He was discharged 5 days after the procedure.

**Figure 3:**
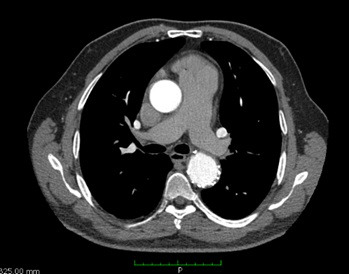
Control angiotomography that demonstrated good stent positioning, excluding the PDA.

At 17 and 45-day follow-up consultations, the patient was in good postoperative recovery. Control angiotomography was performed (Figure 3), showing that the stent was normal in the descending thoracic aorta, with the proximal end close to the orifice of the left subclavian artery, with no signs of endoleak and PDA exclusion.

## DISCUSSION

Many patients begin a diagnostic investigation after auscultation of a heart murmur located on the left sternal border; others are incidentally diagnosed. It is known that asymptomatic patients with moderate PDA may have chronic overload of the left chambers leading to AF and heart failure.^2^^,^^3^^,^^5^ This patient was asymptomatic for 44 years by the time he began to show exercise intolerance. An echocardiography was performed which, in addition to confirming the PDA, already demonstrated the presence of AF and signs of heart failure.

The clinical picture is directly determined by the degree of shunt deviation, from left to right, which depends on the size and length of the PDA and the difference between pulmonary and systemic vascular resistance. Hemodynamic consequences can be categorized by the degree of shunt, based on the ratio of pulmonary to systemic flow (Qp:Qs). Small shunts - Qp:Qs <1.5 – are usually asymptomatic and diagnosed incidentally. Moderate shunts - Qp:Qs between 1.5 and 2.2 - may show intolerance to exercise and an increase in volumetric load of left atrium and ventricle, which leads to ventricular dilatation and dysfunction. In large shunts - Qp: Qs> 2.2 - there is overload of the left ventricular volume, which can progress with increased pulmonary artery pressure, that may cause irreversible pulmonary vascular changes.^12^^,^^13^

Complementary exams for PDA are of fundamental importance. Chest X-ray may be completely normal but can show enlarged pulmonary arteries. The electrocardiogram may be normal or show sinus tachycardia, AF, left ventricular hypertrophy, and enlargement of the left atrium. Echocardiography is the exam of choice to diagnose cases and classify them as silent, small, moderate, or large. It is also useful for assessing other structures and looking for associated malformations. Magnetic resonance imaging or computed tomography (CT) are important tools for visualizing vascular anatomy and the level of arterial calcification. Cardiac catheterization is especially important in adult patients to assess pulmonary vascular resistance and the degree of ductus arteriosus shunt.^2^^,^^5^^,^^12^

**Figure 4:**
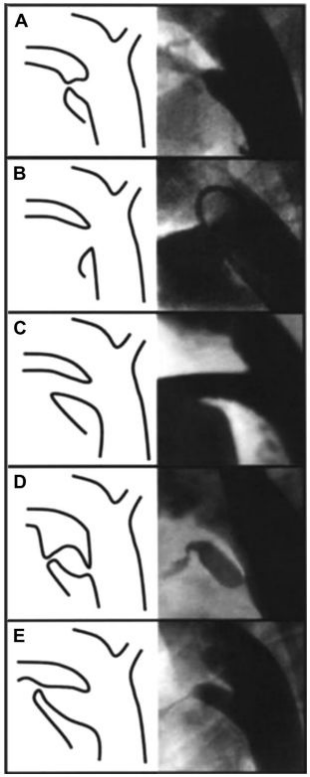
Angiographic classification of ductal anatomy based on the initial description by Krichencko.^2^

PDAs can present in several shapes on angiography. For this reason, Krichenko et al.^14^ classified them into five groups, as illustrated in Figure 4. Type A is a conical duct, with well-defined aortic ampoule and constriction at its pulmonary end; type B is a large and short duct, imitating an aortopulmonary structure similar to a window, which was the case in this patient; type C is a tubular duct with no constriction at its pulmonary end; type D presents with multiple constrictions in the duct; and type E is an elongated duct, often seen in preterm babies.^1^

Current guidelines from the American College of Cardiology, the American Heart Association, and the European Society of Cardiology suggest closure of almost all PDAs, except for small, silent ducts, without audible murmurs and patients with pulmonary arterial hypertension (PAH) with right to left shunt (class III). Class I indications are: (1) shunt associated with volume overload in the left atrium or ventricle; (2) development of PAH, but pressure and resistance still remain below two-thirds of systemic levels; (3) previous history of endarteritis.^13^

The indications for closure of PDA with small and silent shunts are less certain. Since treatment methods are effective and associated with minimal morbidity, a strategy that advocates routine closure of any PDA in children and young adults seems to be reasonable.^1^^,^^13^

Percutaneous and surgical approaches remain the two primary closure methods for adult PDA. Percutaneous closure is the preferred method in adult patients, as surgery carries an increased perioperative risk due to ductal friability, calcification, and associated comorbidities such as coronary artery disease or aortic atherosclerosis.^1^^,^^2^^,^^13^

Transcatheter treatment is now the procedure of choice for most term babies, children, and adults with PDA.^1^^,^^2^^,^^6^^,^^15^ The availability of a variety of devices and techniques increases the ability to treat most patent ducts with catheter-based techniques.^2^

In general, the success rate of Amplatzer duct occlusion is 99% with 76% immediate closure at the time of implantation, 89% success on day 1, and 99% by echocardiography from 6 to 12 months. The “Multicenter USA Amplatzer Patent Ductus Arteriosus Occlusion Device Trial” reported major events in 2.3% of patients, with embolization of the device in 2/439 patients. Other potential complications are flow disturbance in the proximal left pulmonary artery or descending aorta from a protruding device, thrombosis of the femoral artery or vein related to vascular access, and infection.^2^^,^^16^

The Zenith Alpha endoprosthesis is a device for treatment of thoracic aorta pathologies. It was designed to overcome difficult access.^8^^,^^9^ This approach is applicable regardless of the size of the PDA. There are concerns about use of this technique with adults with PDA in patients with small femoral arteries and advanced arteriosclerotic disease and also in patients with anatomical variants of the aortic arch, which can limit effective implantation of the graft.^8^^-^11

Surgery has been successful in managing PDA and is currently the treatment of choice for PDA in preterm infants in whom pharmacological treatment fails. Surgical PDA ligation also remains the treatment of choice for very large ducts or unusual ductal anatomy.^2^^,^^3^^,^^12^

The most feared intraoperative complication is hemorrhage due to injury to the ductus arteriosus near the pulmonary or aortic stump, related to presence of calcification. In these cases, a transpulmonary approach is preferred because the pulmonary stump is less affected by calcifications.^17^

This case demonstrates the natural history of PDA, which varies from asymptomatic to congestive heart failure. The possibility of endovascular treatment using the Zenith Alpha endoprosthesis was presented. This approach can be employed regardless of PDA size and is effective and safe.
